# A new focus of schistosomiasis mansoni in Hayk town, northeastern Ethiopia

**DOI:** 10.1186/s13104-014-0965-z

**Published:** 2015-02-03

**Authors:** Gashaw Amsalu, Zeleke Mekonnen, Berhanu Erko

**Affiliations:** Debre Markos University, P.O. Box 269, Debre Markos, Ethiopia; Department of Medical Laboratory Sciences and Pathology,College of Public Health and Medical Sciences, Jimma University, P.O. Box 378, Jimma, Ethiopia; Aklilu Lemma Institute of Pathobiology, Addis Ababa University, P.O. Box 1176, Addis Ababa, Ethiopia

## Abstract

**Background:**

The endemicity of human schistosomiasis has long been established in Ethiopia, and new foci have also been continuously reported.

The objective of this study was to determine the transmission and magnitude of schistosomiasis in Hayk area, northeastern Ethiopia.

**Methods:**

A cross sectional parasitological survey involving 384 school children was conducted for intestinal schistosomiasis between January and March 2010 in two primary schools in Hayk area, northeastern Ethiopia. The stool samples were processed for microscopic examination using Kato-Katz technique. Malacological survey and observation on human water contact activities were also carried out. Snails were checked for schistosome infection by shedding and lab-bred mice were exposed to the cercariae shed from *Biomphalaria pfeifferi en masse*. Adult *Schistosoma mansoni* worms were harvested from the mice after 45 days of exposure to the schistosome cercariae.

**Results:**

The overall prevalence and intensity of intestinal schistosomiasis among school children in Hayk Number 1 and Hayk Number 2 Primary Schools was found to be 45% and 161 epg, respectively. The prevalence of infection had relationship with age and sex. Males were more infected than females. Children in the age group 15-19 years had the highest infection rate, followed by 10-14 and 5-9 years age group. Schistosome infection in *Biomphalaria pfeifferi* was 3.2%. Schistosome infection was also established in laboratory-bred mice and adult *Schistosoma mansoni* worms were harvested.

**Conclusion:**

The observed intestinal schistosomiasis with prevalence of 45% among young children, collection of schistosome infected *Biomphalaria pfeifferi,* and the establishment of lab infection in mice showed that transmission of intestinal schistosomiasis is taking place in the area. Preventive chemotherapy with praziquantel should be immediately put in place to reduce morbidity and interrupt transmission of schistosomiasis in the area.

## Background

Schistosomiasis is a parasitic disease caused by blood flukes of the genus *Schistosoma*. It is the most important disease in terms of its public health and socioeconomic impact after malaria in many developing countries of the tropics. The burden of the disease is as high as 80-85%, principally in sub-Saharan Africa [1]. Although the majority of the infection is often linked with morbidity, it also results in considerable death. The overall annual mortality rate might exceed 200,000 people in Africa due to different complications of urinary and intestinal schistosomiasis [2]. Children are at a greater risk of acquiring the infection as well as re-infection, and this might cause growth retardation, anemia and low school performance [3,4].

In Ethiopia, human schistosomiasis has been known to be endemic and causes substantial public health and socio-economic impact [5]. As opposed to urogenital schistosomiasis which is limited in distribution and occurring mainly in low lands of Ethiopia, intestinal schistosomiasis is found widely distributed and largely confined to high lands within an altitudinal limit of 800 m-2200 m above sea level. In the last decade, an estimated number of 29.89 million people were at risk, of which 4 million were estimated to be infected in the country [6].

Various epidemiological study results on schistosomiasis are available for different parts of Ethiopia and new transmission foci have also been reported from different parts of the country from time to time. The reasons for the spreading of the disease to new localities seem to be an extensive population movement and water resource development [7]. Additionally, in this era of global warming and climatic change, the epidemiology of temperature-dependent infectious diseases is rapidly changing.

As in many other geographical regions of the country, the endemicity of intestinal schistosomiasis has long been established in Northeastern Ethiopia. For instance, Bati, Borkena River Basin including Harbu and Kemise are endemic for schistosomiasis mansoni [8,9]. The present epidemiological study, therefore, was conducted to determine the transmission and magnitude of intestinal schistosomiasis in Hayk Town, northeastern Ethiopia.

## Methods

### Study area and population

**Figure 1:**
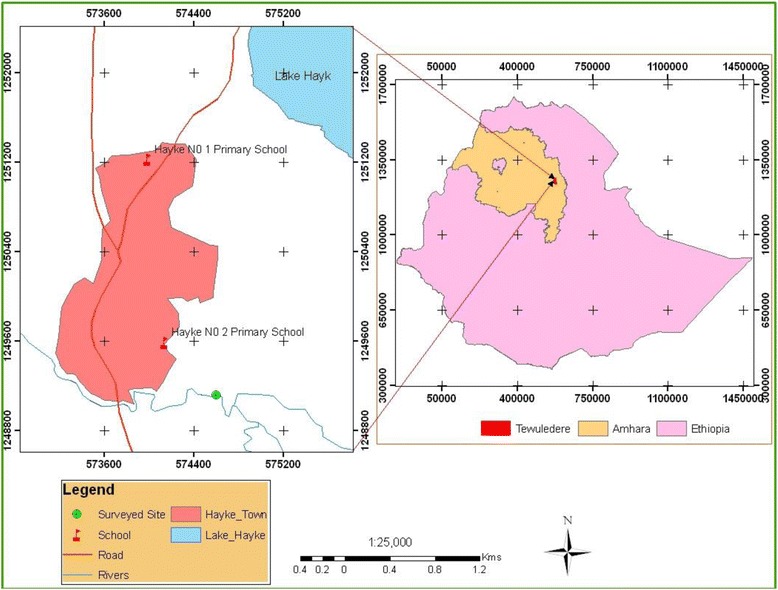
**Map of study site.**

The study was conducted in Hayk Number 1 and Hayk Number 2 Elementary Schools in January and March 2010. The area is located about 430 km from Addis Ababa in the northeast of Ethiopia on the highway from Addis Ababa to Mekele (Figure 1). The area has altitudinal ranges of 1480 to 1900 meters above sea level (masl). The climate is moderately hot with an annual average temperature ranging from 15-21°C and annual rainfall of approximately 1030 mm. The two main fresh water bodies found in the area are Lake Hayk (also called Logo Hayk), covering an area of 23 km^2^ and a permanent stream called Ketie. They are found east and south east of the Hayk Town, respectively. According to the 2007 Population and Housing Census of Ethiopia [10], the population is over 25,000, Muslims being dominant. Hayk Town is an administrative center for many farmers’ associations.

Even though the town dwellers use piped water which greatly reduces the risk of infection by water-borne and water-related diseases, the rural communities had no access to piped water and used the stream or the lake for domestic purposes.

Ketie is a small, shallow and perennial stream. The water discharge of the stream dwindles in the middle of the dry season not only because of a decrease from the source, but also farmers divert water at different sites for small scale irrigation purposes. There are three schools and one health centre in Hayk Town.

The study participants included a total of 384 school children selected from Hayk Number 1(129 children) and Hayk Number 2 (255 children) Primary Schools in Hayk Town in January and March 2010.

### Sample size estimation

The sample size (n) was estimated using the statistical formula [11],

$$ \mathrm{n}=\frac{{\mathrm{z}}^2\;\mathrm{p}\;\left(1-p\right)}{{\mathrm{d}}^2}, $$

Where

- n = sample size
- z = z statistic for a level of confidence (1.96)
- d = precision (0.05)
- p = expected prevalence (0.50)

Since the overall prevalence rate (p) of schistosomiasis was not known for the study area, p was taken to be 50%. For the calculation, a 95% confidence interval (z) and a 5% margin of error (d) were used. This gave a sample size of 384.

The study was cross-sectional and the study participants were selected by systematic sampling using the school master list as a sampling frame. The master lists of the two schools were combined and the total number of children (the population size) was divided by the sample size (384) to determine sampling interval (every 4^th^). Then, every 4^th^ child was selected until the required sample size was reached.

### Stool sample collection and examination

Stool samples were collected from children enrolled at Hayk Number-1and Hayk Number-2 Primary Schools in Hayk Town. Only those children who assented and willing to provide stool, and where the parents consented by signing, participated in the study. Then, those study children were given plastic container with applicator stick and were instructed to bring sizable stool sample of their own. Information including age, sex, and whether the children had been living in Hayk or came from other areas was also recorded at the time of stool collection. The stool samples were processed for microscopic examination using Kato-Katz technique [12]. A single Kato slide (template delivering 41.7 mg of stool)was prepared from stool of each child and the slides were transported to the lab and quantitatively examined for ova of *Schistosoma mansoni* by an experienced laboratory technician within one week of collection. For quality control, 10% of the slides were randomly selected and re-examined by the first author and there was no discrepancy.

Eggs from each Kato slide were multiplied by 24 and infection intensity was expressed as the number of eggs per gram (epg) of faeces. Intensity classes were determined as light, moderate and heavy [13].

### Snail survey

Snails were surveyed both in the Lake Hayk and Ketie Stream at human water contact sites by handpicking using forceps and gloves. The snails collected were transported to Aklilu Lemma Institute of Pathobiology for determination of natural trematode infection. In the case of *Biomphalaria pfeifferi,* the snails were kept in the dark for about 24 hours prior to exposure and then each snail was placed individually in the shedding vials and exposed to electric light for about one hour. Lab-bred mice were exposed to schistosome cercariae shed from *B. pfeifferi* en masse for definite identification of schistosome species, based on egg or adult worm morphology. This was done by immersing the tails of the mice in water containing schistosome cercariae. The exposed mice were maintained in the lab for 8 weeks after which they were sacrificed and adult worms were recovered by perfusion [14].

During snail collection visual observations were also made on physical characteristics of the habitat such as vegetation abundance, turbidity, and the nature of the substrate. Observation was also made on water contact activities and human behavior at selected sites.

### Data analysis

The data were entered into Microsoft Excel, verified and exported to SPSS software version 13 for analysis. Chi-square (*X*^2^) was used to test possible association of infection with sex and age. P-value of less than 0.05 was considered statistically significant. Mean group egg counts were calculated as the geometric mean of all positive children and expressed as eggs per gram (epg) of faeces.

### Ethical considerations

The study obtained ethical clearance from the Institutional Research and Ethics Committee of the Faculty of Life Science, Department of Biology, Addis Ababa University. All children found positive for schistosomiasis were treated with praziquantel in a single oral dose of 40 mg/kg body weight. Children who were found positive for soil-transmitted helminths during stool examination were also treated with a single dose of 400 mg albendazole. Furthermore, the team gave health education to the students about mode of transmission of schistosomiasis and preventive measures to be taken to control the disease.

## Results

### Parasitological survey

The results of stool examination of the two schools are shown in Tables 1 and 2. The prevalence and intensity of intestinal schistosomiasis among school children in Hayk Number 1 Primary School was 13% and 78 epg, respectively, while the respective prevalence and intensity among school children in Hayk Number 2 Primary School was 61% and 179 epg. The difference in prevalence and intensity of infection between the schools is statistically significant (p < 0.05). Both schools combined (n = 384), the overall prevalence and intensity of infection was 45% and 161 epg, respectively. The prevalence and intensity of infection was higher in males than in females for the two schools. The difference in infection rate between males and females is also statistically significant (p < 0.05). Age-wise, children in the 15-19 age group had the highest infection followed by the 10-14, and 5-9 age groups, in that order.

**Table 1:** **Prevalence and intensity of schistosomiasis mansoni among school children in Hayk Number 1 Primary School by age group, Northeastern Ethiopia, 2010**

**Age group**	**% (no. positive/no. examined)**			Eggs per gram of faeces (EPG*)		
	**Female**	**Male**	**Total**	**Female**	**Male**	**Total**
**5-9**	4 (1/25)	13 (4/30)	9 (5/55)	24	48	46
**10- 14**	7 (2/30)	19 (7/37)	13 (9/67)	48	81	80
**15-19**	25 (1/4)	67 (2/3)	43 (3/7)	24	84	64
**Total**	**4 (4/59)**	**19 (13/70)**	**13 (17/129)**	**48**	**81**	**78**

*EPG: geometric mean intensity.

**Table 2:** **Prevalence and intensity of intestinal schistosomiasis among school children in Hayk Number 2 (Ketie) Primary School by age groups, Northeastern Ethiopia, 2010**

**Age group**	**% (no. positive/no. examined)**			Eggs per gram of faeces (EPG*)		
	**Female**	**Male**	**Total**	**Female**	**Male**	**Total**
**5-9**	41(23/56)	59 (42/71)	51 (65/127)	166	191	182
**10-14**	55 (33/60)	82 (51/62)	68 (84/122)	152	187	170
**15-19**	100 (1/1)	100 (5/5)	100 (6/6)	216	240	234
**Total**	**49 (57/117)**	**71 (98/138)**	**61 (155/255)**	**160**	**193**	**179**

*EPG: geometric mean intensity.

During microscopic examination of stool, egg load ranging from 24 to 1608 per gram of stool was recorded for *S. mansoni* infected children. In *S. mansoni* positive children in Hayk Number 1 Primary School, the proportions of light(<100 epg), moderate (100–399 epg), and heavy (≥400 epg) infections were 76%, 18%, and 6% respectively, while the respective proportions of light, moderate and heavy infections among children of Hayk Number 2 Primary School were 52%, 35%, and 13%.

Soil-transmitted helminths observed during stool examination were *Ascaris lumbricoides* (11.5%) and *Trichuris trichiura* (4%). Other rare intestinal parasites (<3%) observed were *Hymenolepis nana*, *Enterobius vermicularis* and *Taenia* species. Since slides were not examined within 30 minutes of stool collection, the status of hookworm infection was not determined.

### Snail survey and laboratory infection of mouse

Live *Biomphalaria pfeifferi* were collected only from Ketie Stream. However, no live snail was found from the Lake Hayk during the survey period, except shells of *B. pfeifferi* and *Melanoides tuberculata* (Mollusca: Thiaridae). Out of 31 *Biomphalaria pfeifferi* collected from Ketie Stream, only one shed (3.2%) schistosome cercariae. *S. mansoni* infection was successfully established in the laboratory mice and adult worms were harvested (two male the remaining females) after eight weeks of laboratory maintenance.

Visual observations on physical characteristics of the stream during snail survey showed that the water was moderately turbid, and the substratum was the mixture of mud and sand. The stream had algal growth and sparse vegetation cover. Observations on human behavior showed that people defecated in open field. The inhabitants also performed such water contact activities as swimming, bathing, playing in water, cutting grass, drawing water, performing religious rites such as wadu by Muslims, washing clothes and horses. Swimming and playing in the stream were water contact activities performed by children. The land surrounding the stream is arable and farmers draw water for watering of vegetables such as carrot.

## Discussion

In this study, both the results of parasitological studies and snail survey showed that transmission of intestinal schistosomiasis is taking place in Ketie Stream in Hayk Town, northeastern Ethiopia. The respective intestinal schistosomiasis prevalence of 13% and 61% observed among school children in Hayk Number 1 and Hayk Number 2 Primary Schools, collection of schistosome-infected *Biomphalaria pfeifferi* from Ketie Stream, and establishment of schistosome infection in lab-bred mice all confirmed that transmission of intestinal schistosomiasis is well established in the study area. Since there was no previous report for schistosomiasis transmission in the area, it is difficult to speculate whether schistosomiasis has existed in the area for undetermined period of time or has recently been introduced as a result of ecological changes and population movements. Nevertheless, since the study area is not an isolated one, it is unlikely that schistosomiasis has been there from time immemorial. Although the presence of schistosomiasis cases in an area does not neccessarily show the establishment of an autochthonous transmission, the records of Hyak Health Center showed that schistosome infections were uncommon in the day-to-day routine stool examination. Thus, it is reasonable to assume that the disease is of recent introduction as a result of ecological changes and population movement.

The observed differences in prevalence and intensity of schistosomiasis between the two schools, i.e., higher in Hayk Number 2 than in Hayk Number 1 could be explained by distance of the schools from the Ketie Stream where transmission is taking place. Hayk Number 2 Primary School is in close proximity to the stream (about 1 km), while Hayk Number 1 is a bit far from the stream (about 2.5 km). Hence, the chance for school children of Hayk Number 2 Primary School would be high to perform frequent water contact activities and acquire heavy infection. Moreover, this observation reflects the focal nature of schistosome infection and transmission [15].

The observation that the highest proportion of the infected children had light infection (76%), followed by moderate (18%) and the least heavy infections (6%) reflects the general pattern of schistosome infection. Anderson and May [16] described that intestinal worm burdens exhibit a highly aggregated or overdispersed distribution that most individuals harbor just a few worms, while a few hosts harbor disproportionately large worm burdens. The overdispersion of worm population among the infected children has important epidemiological and public health implications; heavily infected individuals are at the same time at highest risk of morbidity and the major source of environmental contamination [17]. According to Hotez and his colleagues [17], the underlying cause of such predisposition may be attributable to factors such as exposure to infection or differences in susceptibility to infection and the ability to mount effective immunity, among others. Hence, targeting of chemotherapy to school age children, population segment that contains heavily infected individuals, has the two-fold advantage of eliminating morbidity and blocking transmission.

The prevalence and intensity of schistosomiasis was found to be higher in males than in females in the two schools studied. This observation agrees with several previous reports from different parts of Ethiopia including the reports by Birrie [8] from Borkena River Basin, Legese et al. [18] from Adwa Town, Dejenie *et al*. [19] from Waja in Tigray and Erko and Tedla [20] from Zeghie Peninsula of Lake Tana. Similarly, Goselle et al. [21] and Mudathir et al. [22] reported higher prevalence of infection among males in Nigeria and the Sudan, respectively. Systematic water contact studies were not conducted in the present investigation, higher infection rates in male children might be attributable to more frequent water contact patterns than in females. Generally, male children tend to spend more time playing, swimming and bathing in water. Hence, the higher chance is for male children to acquire more infection and re-infection.

In this study, the prevalence of schistosomiasis also tended to rise with age. The lowest prevalence of infection was observed for the 5-9 age group. This agrees with previous reports in other schistosomiasis endemic foci of Ethiopia [9,20,23]. John *et al.* [24] also reported similar patterns of infections with age in Uganda.

The low infection rate in the 5-9 age group is explained by lower water contact activities compared with children in their second decade of life.

Out of the 31 *Biomphalaria pfeifferi* collected, only one (3.2%) snail was found infected with schistosome cercariae. As Anderson and May [16] suggested, high rates of infected snail mortality and the duration of the latent period of infection within the snail are closely associated and accounted for the low infection rate in snail populations. Nevertheless, cercariae shed by a single infected snail would suffice to infect considerable number of people because schistosome multiplication in snail intermediate hosts is enormous.

Visual observations made on the physical characteristics of Ketie Stream revealed that the habitat is highly favorable for *Biomphalaria pfeifferi* and other planorobid snails. The stream was little turbid, slow flowing, had vegetation cover, and support algal growth. It has been described that small streams with flow rate of 10–30 cm/s, with slight turbidity, abundant vegetation and muddy bottoms have been found to be potentially favorable habitats for *B. pfeifferi* and other snails including *Bulinus truncatus* and *Lymnaea natalensis* [25]. In addition, human contaminative activities such as open air defecation and exposure activities such as swimming, bathing, and laundering were conducive for increased transmission of schistosomiasis in the area. Alebie et al. [26] also observed similar contaminative and exposure activities in Sanja area, Amhara Regional State of Ethiopia

## Conclusions

In conclusion, the present observations revealed that the transmission of intestinal schistosomiasis due to *Schistosoma mansoni* has been well established in Ketie Stream as evidenced by observation of high prevalence of infection in school children, collection of schistosome-infected *Biomphalaria pfeifferi* from Ketie Stream, and establishment of schistosome infection in lab-bred mice. Immediate treatment of all school age children and adults at higher risk is recommended once a year [27] until the disease is of no longer public health importance. Other measures such as snail control, health education, improved access to clean water and sanitation should also complement chemotherapy to maintain the low level of morbidity and transmission achieved by drug treatment until schistosomiasis becomes eradicated from the area.
